# Error Analysis for RADAR Neighbor Matching Localization in Linear Logarithmic Strength Varying Wi-Fi Environment

**DOI:** 10.1155/2014/647370

**Published:** 2014-02-09

**Authors:** Mu Zhou, Zengshan Tian, Kunjie Xu, Xiang Yu, Haibo Wu

**Affiliations:** ^1^Chongqing Key Lab of Mobile Communications Technology, Chongqing University of Posts and Telecommunications, Chongqing 400065, China; ^2^Department of Electronic and Computer Engineering, The Hong Kong University of Science and Technology, Kowloon, Hong Kong; ^3^Graduate Telecommunications and Networking Program, University of Pittsburgh, Pittsburgh, PA 15260, USA; ^4^China Internet Research Lab, China Science and Technology Network, Computer Network Information Center, Chinese Academy of Sciences, Beijing 100190, China

## Abstract

This paper studies the statistical errors for the fingerprint-based RADAR neighbor matching localization with the linearly calibrated reference points (RPs) in logarithmic received signal strength (RSS) varying Wi-Fi environment. To the best of our knowledge, little comprehensive analysis work has appeared on the error performance of neighbor matching localization with respect to the deployment of RPs. However, in order to achieve the efficient and reliable location-based services (LBSs) as well as the ubiquitous context-awareness in Wi-Fi environment, much attention has to be paid to the highly accurate and cost-efficient localization systems. To this end, the statistical errors by the widely used neighbor matching localization are significantly discussed in this paper to examine the inherent mathematical relations between the localization errors and the locations of RPs by using a basic linear logarithmic strength varying model. Furthermore, based on the mathematical demonstrations and some testing results, the closed-form solutions to the statistical errors by RADAR neighbor matching localization can be an effective tool to explore alternative deployment of fingerprint-based neighbor matching localization systems in the future.

## 1. Introduction

Motivated by the increasing interests in the location-based ubiquitous computing and context-awareness in the future heterogeneous wireless personal networks (WPN), the seamless and accurate localization systems have caught significant attention in the recent decade [1, 2]. Although the widely-used Global Positioning System (GPS) and cellular networks (e.g., E911) can provide enough accuracy for the existing location-based services (LBSs) in the outdoor environments [3, 4], the performance could be seriously deteriorated in the indoor or underground environments owing to the unavailability of locating signals which are always blocked by the buildings or grounds [5, 6].

To solve this problem, the world's first Wi-Fi fingerprint-based localization system for the indoor environments, the RADAR [7], was proposed by the Microsoft Research in the year 2000. After that, an increasing number of universities and institutes began to study the indoor accurate and real-time neighbor matching localization [8–15]. In these works, the indoor straight corridor scenario with the linearly-calibrated reference points (RPs) is selected as the test-bed due to the simple received signal strength (RSS) propagation characteristic [16] and the purposes of people's path navigation and activity learning in target area [8]. Nowadays, the most representative indoor localization systems are Carnegie Mellon's CMU-PM and CMU-TMI [9]; MIT's Cricket which has provided a practical solution to the improvement of localization scalability, privacy, and agility [10]; Bayesian network-based Nibble localization system which relies on the signal to noise ratio (SNR) to conduct the position matching [11]; Maryland's Horus which has been recognized as the archetype of the fingerprint-based probabilistic localization [12]; and RWTH Aachen University's Markov localizer [13]. Among them, the Wi-Fi fingerprint-based neighbor matching localization (e.g., the RADAR) is addressed as one of the best approaches to perform the position matching by the reasons of low infrastructure and device cost and free license to access 2.4 GHz ISM band [14, 15].

The RADAR neighbor matching localization contains the offline phase (or the site-survey phase) and the online phase (or the localization phase) [7]. More specifically, in the offline phase, we place several access points (APs) in target area to provide the sufficient RSS coverage and also record the RSS fingerprints at each calibrated RP to construct a radio map corresponding to the target area. The radio map can be described as the mapping relations between the RSS distributions and the location coordinates. Then, in the online phase, for the newly recorded RSSs, we conduct the position matching by locating the target's position at the geometric center of the *K* nearest neighboring RPs (or the neighbors). The RPs are regarded as the neighbors if their RSS fingerprints have the smallest distances to the newly-recorded RSSs.

The major contribution of this paper is that we derive out the closed-form analytical result of the statistical errors by RADAR neighbor matching localization. With these solutions, we can answer the following two questions: (i) how can the statistical errors vary with respect to the number and interval of RPs? And (ii) how can we obtain the optimized deployment of RPs to achieve the smallest statistical errors?

This paper is organized as follows. In Section 2, we present some related works on the neighbor matching localization in indoor Wi-Fi environment. In Section 3, we show the detailed analytical derivation for the closed-form solutions to the statistical errors by RADAR neighbor matching localization. In Section 4, the numerical and experimental results are provided to verify the analytical results in Section 3. Finally, we conclude this paper and also address some future directions in Section 5.

## 2. Related Work

As the most representative neighbor matching localization system, the RADAR [7] utilizes the *K* nearest neighbor (KNN) algorithm to infer target's position in the buildings. With the assumption that the recorded RSS is decreased with its distance to the AP, the RADAR system is considered as a deterministic geometrical approach for the position matching [17].

The Horus [12] has been recognized as one of the most sophisticated probabilistic matching localization systems. With the help of joint clustering technique, Horus can achieve the higher accuracy of 90 percent within 2 m compared to the RADAR. Moreover, with the technique of Bayesian inference and decision, the increasing number of fingerprints can further improve estimation for the RSS means and deviations and results in higher accuracy of the probabilistic matching localization.

Similar to the RADAR, the authors in [18] provided an efficient solution to reduce the cost in the offline phase. In many large-scale or difficult-to-access areas, the RSS fingerprint recording seems to be unpractical and unfeasible. To solve this problem, instead of recording the whole fingerprints, only a small number of RPs are selected for the fingerprint recording, while the complete radio map is derived by using the RSS propagation models corresponding to the target area.

The neural network has also been suggested as an effective way to perform the position matching. The authors in [19–22] proposed the neural network-based classifiers to infer the target's coordinates. As discussed in [19], the neural network can achieve an accuracy of 70 percent within 1 m errors which is higher than the deterministic and probabilistic approaches in [7, 12]. However, the major disadvantage of the neural network-based localization is that the accuracy heavily relies on the classifier training. In [20], the one-step secant training with multilayer perceptron architecture has been considered for the training process. Other similar work on neural network-based neighbor matching localization can be found in [21, 22].

The authors in [23] addressed another concept of neighbor matching localization. Instead of locating the target at the precise coordinates, the objective is to infer the subarea where the target is most likely to be located. For each AP, we record the AP's location and MAC address and also its coverage range (or the covered subareas) during the offline phase. Then, in the online phase, with respect to each localization request, we conduct the neighbor matching to the subareas corresponding to the hearable APs by the receiver. Stemmed from this idea, a variety of real-time locating system (RTLS) devices have been developed by Ekahao company [24]. By using the tags attached to the target to measure the RSSs from hearable APs, the Ekahao devices have achieved the accuracy with an error of 3 meters in practical use.

## 3. Statistical Errors by RADAR Neighbor Matching Localization

### 3.1. RADAR Localization

The RADAR system provides the basic model for the neighbor matching localization, as described in the following:

(1) $$\begin{array}{c} C^{*}=\frac{\sum_{j=1}^{K}R_{j}}{K}=\left(\frac{\sum_{j=1}^{K}x_{j}}{K},\frac{\sum_{j=1}^{K}y_{j}}{K}\right),\;\;R_{j}=\left(x_{j},y_{j}\right), \end{array}$$

where C* is the estimated position of test point (TP); {**R**_*j*_ = (*x*_*j*_, *y*_*j*_) : *j* = 1,…, *K*} is the set of neighbors; (*x*_*j*_, *y*_*j*_) is the 2-dimentional coordinates of **R**_*j*_; and *K* is the number of neighbors. In (1), we can find that C* is calculated from the number and coordinates of neighbors (i.e., *K* and (*x*_*j*_, *y*_*j*_)). Therefore, in this paper, we focus on the mathematical relations between the statistical errors and deployment of RPs for the RADAR neighbor matching localization. The other parameters and notations for the following analysis are listed in Parameters and Notations section.

### 3.2. Linear RP Calibration Model

**Figure 1:**
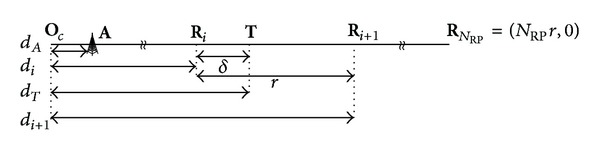
Deployment of RPs in linear calibration model.

The basic linear RP calibration model discussed in this paper is shown in Figure 1. In this model, the *N*_RP_ RPs are assumed to be uniformly calibrated in a linear target area with the same interval *r*. The distances from the origin **O**_c_ to the AP **A** and TP **T** are *d*_**A**_ (0 ≤ *d*_**A**_ < *d*_*i*_) and *d*_*i*_ + *δ* (0 ≤ *δ* ≤ *r*). For simplicity, we assume that the means of received power at **R**_*i*_ and **T** can be calculated by a simple logarithmic RSS propagation model (i.e., *P*_*i*_ = *P*_*t*_ − *L*_0_ − 10*α*log⁡_10_⁡(*d*_*i*_ − *d*_**A**_) and *P*_**T**_ = *P*_*t*_ − *L*_0_ − 10*α*log⁡_10_(*d*_**T**_ − *d*_**A**_), where *L*_0_ and *α* stand for the path loss in the first meter and the path loss exponent, resp.).

In concrete terms, we calculate the statistical errors by RADAR localization following the two main steps below: (i) deducing the closed-form solutions to the errors with the assumption that the real position of the TP is at **T** = (*d*_*i*_ + *δ*, 0) and (ii) calculating the expectation of the closed-form errors with respect to *δ* and *d*_*i*_.

### 3.3. Statistical Errors by One-Neighbor Matching Localization

Based on the logarithmic RSS propagation model, in this situation, C* can only be located at **R**_*i*_ or **R**_*i*+1_ which has the smallest RSS difference to the mean of received power at **T**. Therefore, we have two cases to be discussed as follows.

#### 3.3.1. Case 1: *d*_**T**,1_ =  *d*_*i*_ with Error *δ*

To locate C* at **R**_*i*_, the relations *P*_*i*_ − *P*_**T**_ ≤ *P*_**T**_ − *P*_*i*+1_ should be satisfied. Therefore, the mathematical relations between *δ* and *d*_**A**_ can be described as

(2) 0≤δ<r2, if  and  only  if  0≤dA≤di−δ2(r−2δ);r2<δ≤r, if  and  only  if  di−δ2(r−2δ)<dA≤di;δ=r2, if  and  only  if  dA∈∅.

Equation (2) can be simplified as

(3) 0≤δ<r2, if  and  only  if  0≤δ≤di2+dir−di<r2;r2<δ≤r, if  and  only  if  dA∈∅.

Based on (2) and (3), one has

(4) $$\begin{array}{c} 0\leq \delta \leq \sqrt{d_{i}^{2}+d_{i}r}-d_{i},\;\text{if}\;\text{and}\;\text{only}\;\text{if}\;0\leq d_{A}\leq \frac{d_{i}-\delta^{2}}{\left(r-2\delta \right)}. \end{array}$$

At this point, by assuming that *δ* satisfies the uniform distribution in the range of $\left(0,\sqrt{d_{i}^{2}+d_{i}r}-d_{i}\right)$, we can calculate

(5) Eδ{di−δ2(r−2δ)}=Eδ{di}−Eδ{δ2(r−2δ)}=di−1di2+dir−di ×∫0di2+dir−diδ2(r−2δ) dδ=di+ℵ4+r4−r2ln⁡r/(r−2ℵ)8ℵ,

where $ℵ=\sqrt{d_{i}^{2}+d_{i}r}-d_{i}$. Then, we continue to calculate the expectation with respect to *d*_*i*_ to obtain the confidence probability for this case (i.e., Prob_1,1_):

(6) Prob1,1=Edi{di+ℵ4+r4−r2ln⁡r/(r−2ℵ)8ℵ}=1NRP∑i=1NRP(1+ℵ4di+r4di−r2ln⁡r/(r−2ℵ)8ℵdi).

By Taylor expansion of $\sqrt{d_{i}^{2}+d_{i}r}$ in (7), Prob_1,1_ can be simplified into (8):

(7) di2+dir=di(1+rdi)1/2=di(1+12(rdi)−1×12×4(rdi)2 +1×1×32×4×6(rdi)3 −1×1×3×52×4×6×8(rdi)4+⋯)=di(1+12(rdi)−18(rdi)2 +116(rdi)3+O(1di)4), |r|≤|di|,

(8) Prob1,1=1+(r8NRP∑i=1NRP1di+O(1di)2) +(r4NRP∑i=1NRP1di) −(r2NRPln⁡r2∑i=1NRP1di −r4NRP∑i=1NRPln⁡didi+O(1di)2)=1+18NRP(3+4ln⁡r2)∑i=1NRP1i −14NRP∑i=1NRPln⁡iri+O(1di)2=1+38NRP∑i=1NRP1i+14NRP ×(ln⁡r4∑i=1NRP1i−∑i=1NRPln⁡ii −∑i=1NRPln⁡ri)+O(1di)2=1+18NRP(3−4ln⁡2)∑i=1NRP1i −14NRP∑i=1NRPln⁡ii+O(1di)2.

#### 3.3.2. Case 2: *d*_**T**,1_ = *d*_*i*+1_ with Error *r* − *δ*

Owing to the complementary property, the confidence probability in this case (i.e., Prob_1,2_) can be simply calculated by the following:

(9) Prob1,2=1−Prob1,1=14NRP∑i=1NRPln⁡ii−18NRP(3−4ln⁡2)∑i=1NRP1i+O(1di)2.

Therefore, the expected error by RADAR one-neighbor matching localization (i.e., *ε*_1_) can be calculated by

(10) ε1=Prob1,1ε1,1+Prob1,2ε1,2=Prob1,1Edi{Eδ{δ}}+Prob1,2(r−Edi{Eδ{δ}})=Prob1,1Edi{1ℵ∫0ℵδ dδ} +Prob1,2(r−Edi{1ℵ∫0ℵδ dδ})=Prob1,1(r4−r216NRP∑i=1NRP1di+O(1di)2) +Prob1,2(3r4+r216NRP∑i=1NRP1di+O(1di)2)=r4+r8NRP∑i=1NRPln⁡iri+O(1di)2≈r4+r8NRP∑i=1NRPln⁡iri,

where *ε*_1,1_ and *ε*_1,2_ stand for the expected errors in Case 1 and Case 2, respectively.

### 3.4. Statistical Errors by Two-Neighbor Matching Localization

From the logarithmic RSS propagation model, the distance between **O**_c_ and C* can only be (*d*_*i*_ + *d*_*i*+1_)/2 or (*d*_*i*+1_ + *d*_*i*+2_)/2. Therefore, we have the following two cases to be discussed in this situation.

#### 3.4.1. Case 1: *d*_**T**,2_ = (*d*_*i*_ + *d*_*i*+1_)/2 with Error |*δ* − *r*/2|

In this case, to satisfy the relations of *P*_*i*_ − *P*_**T**_ ≤ *P*_**T**_ − *P*_*i*+2_, we should require

(11) 0≤δ<r, if  and  only  if  0≤dA≤di−δ22(r−δ);δ=r, if  and  only  if  dA∈∅.

By calculating (11), one obtains

(12) 0≤δ<r, if  and  only  if  0≤δ≤di2+2dir−di<r;δ=r, if  and  only  if  dA∈∅.

From (11) and (12), we have

(13) $$\begin{array}{c} 0\leq \delta \leq \sqrt{d_{i}^{2}+2d_{i}r}-d_{i},\;\text{if}\;\text{and}\;\text{only}\;\text{if}\;0\leq d_{A}\leq \frac{d_{i}-\delta^{2}}{2\left(r-\delta \right)}. \end{array}$$

Similarly, we assume that the *δ* satisfies the uniform distribution in the range of $\left(0,\sqrt{d_{i}^{2}+2d_{i}r}-d_{i}\right)$. Then, we can calculate

(14) Eδ{di−δ22(r−δ)}=Eδ{di}−Eδ{δ22(r−δ)}=di−1di2+2dir−di ×∫0di2+2dir−diδ22(r−δ)dδ=di+Θ4+r2−r22Θln⁡rr−Θ,

where $\Theta =\sqrt{d_{j}^{2}+2d_{j}r}-d_{j}$. Then, the confidence probability for this case (i.e., Prob_2,1_) can be obtained by calculating the expectation with respect to *d*_*i*_ in the following:

(15) Prob2,1=Edi{di+Θ4+r2−r22Θln⁡rr−Θ}=1NRP∑i=1NRP(1+Θ4di+r2di−r22Θdiln⁡rr−Θ).

Using the Taylor expansion of $\sqrt{d_{j}^{2}+2d_{j}r}\left(\left|2r\right|\leq \left|d_{j}\right|\right)$ in (16), we can simplify (15) into (17):

(16) di2+dir=di(1+2rdi)1/2=di(1+12(2rdj)−18(2rdj)2 +116(2rdj)3+O(1di)4),

(17) Prob2,1=1+(r4NRP∑i=1NRP1di+O(1di)2) +(r2NRP∑i=1NRP1di) −(r2NRPln⁡r2∑i=1NRP1di −r2NRP∑i=1NRPln⁡didi+O(1di)2)=1+14NRP(3+2ln⁡r2)∑i=1NRP1i −12NRP∑i=1NRPln⁡iri+O(1di)2=1+34NRP∑i=1NRP1i +12NRP(ln⁡r2∑i=1NRP1i−∑i=1NRPln⁡ii −∑i=1NRPln⁡ri)+O(1di)2=1+14NRP(3−2ln⁡2)∑i=1NRP1i −12NRP∑i=1NRPln⁡ii+O(1di)2.

#### 3.4.2. Case 2: *d*_**T**,2_ = (*d*_*i*+1_ + *d*_*i*+2_)/2 with Error |*δ* − 3*r*/2|

Due to the complementary of probability, the confidence probability for this situation (i.e., Prob_2,2_) is equal to

(18) Prob2,2=1−Prob2,1=12NRP∑i=1NRPln⁡ii−14NRP(3−2ln⁡2)∑i=1NRP1i+O(1di)2.

Therefore, from (17) and (18), the expected error by RADAR localization in the two-neighbor matching situation (i.e., *ε*_2_) is calculated in the following:

(19) ε2=Prob2,1ε2,1+Prob2,2ε2,2=Prob2,1Edi{Eδ{|δ−r2|}} +Prob2,2(r−Edi{Eδ{|δ−3r2|}})=Prob2,1Edi{(1Θ∫0r/2(r2−δ)dδ +1Θ∫r/2Θ(δ−r2)dδ)} +Prob2,2(3r2−(1Θ∫0r/2(r2−δ)dδ +1Θ∫r/2Θ(δ−r2)dδ))=Prob2,1(r4−r24NRP∑i=1NRP1di+O(1di)2) +Prob2,2(5r4+r24NRP∑i=1NRP1di+O(1di)2)=r4+r2NRP∑i=1NRPln⁡iri+O(1di)2≈r4+r2NRP∑i=1NRPln⁡iri,

where *ε*_2,1_ and *ε*_2,2_ stand for the expected errors in Case 1 and Case 2, respectively.

### 3.5. Statistical Errors by Multineighbor Matching Localization

In this subsection, we prove that the expected errors by multineighbor matching localization are larger than the errors in the one- and two-neighbor matching situations by using our proposed linear RP calibration model.

Without losing generality, we assume that the *K* neighbors used for the neighbor matching localization are at **R**_*ℓ*_ (*ℓ* = *j* − *N*_1_ + 1, *j* − *N*_1_ + 2,…, *j* + *N*_2_; *N*_1_ + *N*_2_ = *K*) in the linear RP calibration model. Then, by (1), the errors in *K*-neighbor matching situations can be easily calculated in the following:

(20) εK=|(di+δ)−dT,K|=|(di+δ)−(di+N2−N1+12r)|   =|δ−ξ2r|,ξ=N2−N1+1.

As can be seen in (20), we can find that (i) by one-neighbor matching localization, we have the relations of “*N*_1_ = 1  and  *N*_2_ = 0 (i.e., *ξ* = 0)” or “*N*_1_ = 0  and  *N*_2_ = 1 (i.e., *ξ* = 2).” Then, one has *ε*_1_ = |*δ*| or *ε*_1_ = |*δ* − *r*|; (ii) by two-neighbor matching localization, we have the relations of “*N*_1_ = 1 and *N*_2_ = 1 (i.e., *ξ* = 1)” or “*N*_1_ = 0  and  *N*_2_ = 2 (i.e., *ξ* = 3).” Then, one has *ε*_2_ = |*δ* − *r*/2| or *ε*_2_ = |*δ* − 3*r*/2|; and (iii) by multineighbor matching localization, one has *ε*_*K*_ ≥ |*δ* + *r*/2| > |*δ*| = *ε*_1_ and *ε*_*K*_ ≥ |*δ* + *r*/2| ≥ |*δ* − *r*/2| = *ε*_2_ when *ξ* ≤ −1, while in the condition of *ξ* ≥ 4, one has *ε*_*K*_ ≥ |*δ* − 2*r*| = 2*r* − *δ* ≥ |*δ*| = *ε*_1_ and *ε*_*K*_ ≥ |*δ* − 2*r*| = 2*r* − *δ* > |*δ* − *r*/2| = *ε*_2_. Therefore, the expected errors in one- and two-neighbor matching situations are proved to be smaller than the errors by multineighbor matching localization in the linear RP calibration model, by assuming that the mean of received power is estimated by the logarithmic RSS propagation model. Finally, the closed-form result of the expected errors by RADAR neighbor matching localization in one- and two-neighbor matching situations is shown in Table 1. For simplicity, the higher order terms are not included in the table.

### 3.6. Variations of Statistical Errors

The previous discussion on the statistical errors by RADAR neighbor matching localization is with the assumption that the mean of received power at both the RPs and TP can be estimated by the logarithmic RSS propagation model [25]. However, in many mobile scenarios (e.g., the motion tracking), we cannot record the RSSs at a fixed TP for a long time to obtain the precise estimation of the mean power [26, 27]. In other words, the real-time values of RSS at the TP could vary dramatically compared to the mean power which is calculated from the logarithmic RSS propagation model. Thus, we present some preliminary results on the variations of expected errors with respect to the testing RSSs. For the sake of simplicity, we only focus on the one- and two-neighbor matching situations with the three closest neighbors **R**_*i*_, **R**_*i*+1_, and **R**_*i*+2_.

#### 3.6.1. Error Variations by One-Neighbor Matching Localization

We assume that **R**_*i*_ is the neighbor which has the largest confidence probability when there is no variation on the testing RSS. In other words, the testing RSS is obtained from the logarithmic RSS propagation model. Then, if the RSS variations at the TP occur, the confidence probability of **R**_*i*_ decreases into Prob_1,1_′ = Prob_1,1_ − *λ*_1,2_ − *λ*_1,3_, where *λ*_1,2_ and *λ*_1,3_ stand for the increased percentages for the probabilities of **R**_*i*+1_ and **R**_*i*+2_ (i.e., Prob_1,2_′ = Prob_1,2_ + *λ*_1,2_ and Prob_1,3_′ = *λ*_1,3_), respectively. Therefore, the expected error (i.e., *ε*_1_′) and the corresponding increasing rate of error (i.e., *ρ*(*ε*_1_)) in this situation become

(21) ε1′=Prob1,1′ε1,1+Prob1,2′ε1,2+Prob1,3′ε1,3=(Prob1,1−λ1,2−λ1,3)ε1,1 +(Prob1,2+λ1,2)ε1,2+λ1,3(2r−ε1,1)=ε1+(λ1,2+λ1,3)(r−2ε1,1)+rλ1,3>ε1,ρ(ε1)=((λ1,2+λ1,3)(r−2ε1,1)+rλ1,3)ε1.

#### 3.6.2. Error Variations by Two-Neighbor Matching Localization

In this situation, the probability of selecting **R**_*i*+1_ and **R**_*i*+2_ as the two neighbors could be calculated as Prob_2,2_′ = Prob_2,2_ + *λ*_1,2_ + *λ*_1,3_ when the RSS variations at the TP occur. Then, based on the complementary property, the probability for neighbors **R**_*i*+1_ and **R**_*i*+2_ decreases into Prob_2,1_′ = Prob_2,1_ − *λ*_1,2_ − *λ*_1,3_. Therefore, we obtain the expected error (i.e.,  *ε*_2_′) and the corresponding increasing rate of error (i.e., *ρ*(*ε*_2_)) as follows:

(22) ε2′=Prob2,1′ε2,1+Prob2,2′ε2,2=(Prob2,1−λ1,2−λ1,3)ε2,1+(Prob2,2+λ1,2+λ1,3)ε2,2=ε2+(λ1,2+λ1,3)(32r−2ε2,1)>ε2,ρ(ε2)=(λ1,2+λ1,3)(3r/2−2ε2,1)ε2.

## 4. Numerical and Experimental Results

### 4.1. Numerical Results

**Figure 2:**
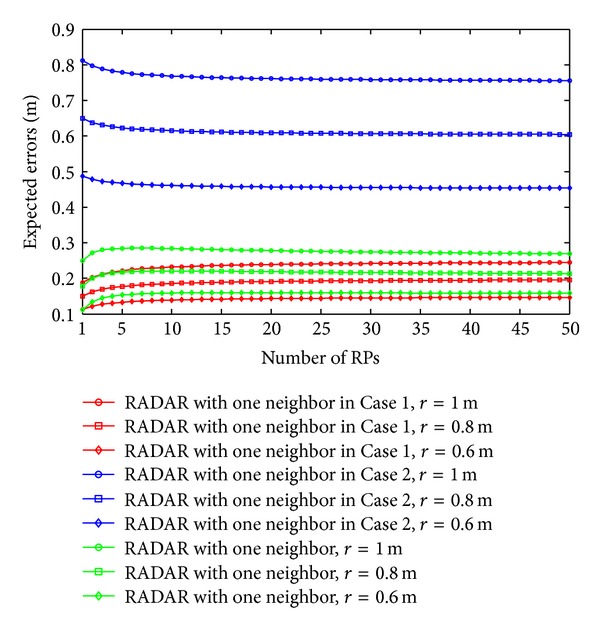
Expected errors by one-neighbor matching localization.

**Figure 3:**
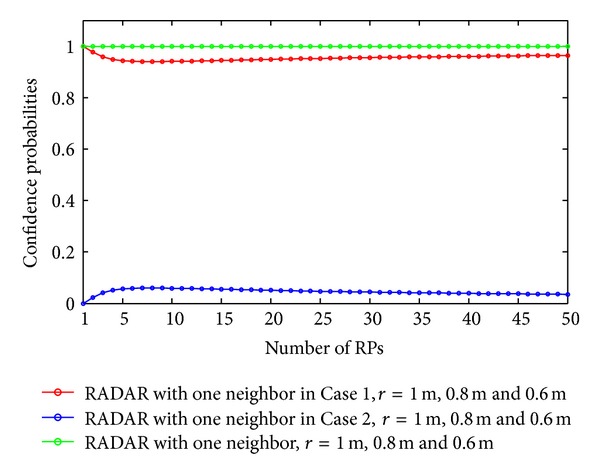
Confidence probabilities by one-neighbor matching localization.

**Figure 4:**
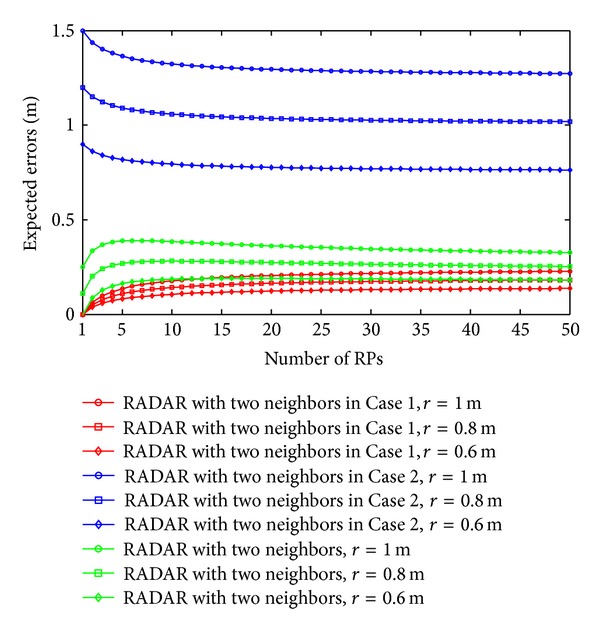
Expected errors by two-neighbor matching localization.

**Figure 5:**
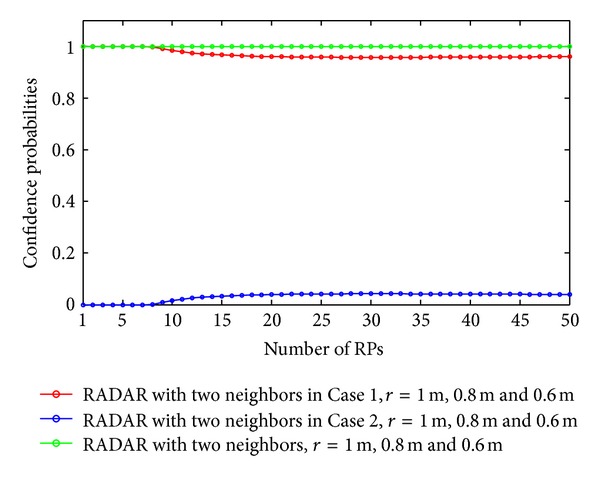
Confidence probabilities by two-neighbor matching localization.

As discussed in our previous section, the expected errors of the RADAR neighbor matching localization significantly depend on the deployment of RPs. In Figures 2, 3, 4, and 5, we show the variations of expected errors and the related confidence probabilities by one- and two-neighbor matching localization with respect to the number and interval of RPs (i.e., *N*_RP_ and *r*).

**Figure 6:**
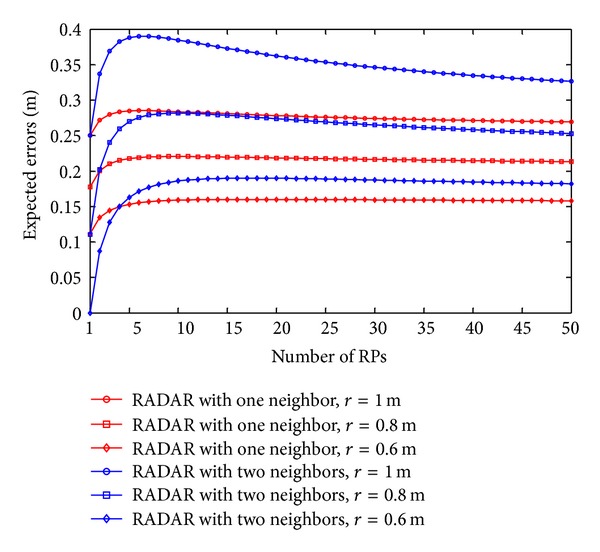
Error comparison by one- and two-neighbor matching localization.

From the previous figures, we can observe that, in both the one- and two-neighbor matching situations, (i) the expected errors decrease with the reduction of distance between two adjacent RPs and (ii) the expected errors become stable when the dimensions of the target area are large enough (e.g., *N*_RP_ *r* ≥ 10 m). In other words, both the one- and two-neighbor matching localization can be applied to the large-scale target area, while preserving the localization accuracy. To examine the errors between these two situations further, Figure 6 compares the errors by one- and two-neighbor matching localization.

In Figure 6, the one-neighbor matching localization achieves higher accuracy, especially in the condition of large *r*. The significant decrease of expected errors by two-neighbor matching localization in small *N*_RP_ condition is mainly because the geometrical center of the small-scale target area (e.g., *N*_RP_ *r* ≤ 5 m) can be suggested as a reasonable location estimation for the target irrespective of its real position in this area, while the increasing number of RPs can provide the better estimation for this geometric center. Furthermore, the tradeoff between the RP calibration effort and the accuracy improvement by RADAR neighbor matching localization is another contribution of this paper. Let us take the condition of *N*_RP_ *r* ≥ 40 m as an example. When *r* decreases from 1 m to 0.8 m and then to 0.6 m, the variations of the number of RPs and expected errors are shown in Table 2. As we can see from Table 2, in the one-neighbor matching situation, when *r* decreases from 0.8 m to 0.6 m, we have more than 40 percent growth in the number of required RPs, but less than 20 percent drop in the expected errors.

**Figure 7:**
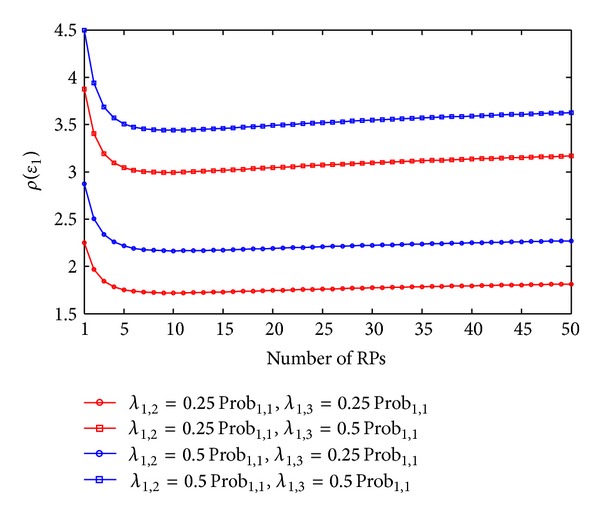
Variations of increasing rates of errors by one-neighbor matching (*r* = 1 m).

**Figure 8:**
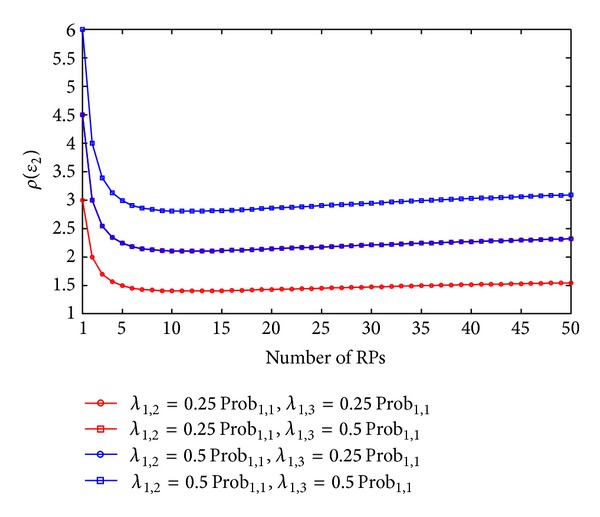
Variations of increasing rates of errors by two-neighbor matching (*r* = 1 m).

Finally, Figures 7 and 8 illustrate the variations of expected error by one- and two-neighbor matching localization. In Figures 7 and 8, we can observe that in both one- and two-neighbor matching situations (i) the increasing rates of errors increase with the variations of testing RSSs (or *λ*_1,2_ + *λ*_1,3_). In other words, the errors by RADAR neighbor matching localization are vulnerable to the variations of RSSs at the TP and (ii) in the small-scale target area (e.g., *N*_RP_ *r* ≤ 5 m), the increasing rates of errors increase dramatically with the decrease of value *N*_RP_ *r*, while the increasing rates of errors change gently in the large-scale target area (e.g., *N*_RP_ *r* ≥ 10 m). Moreover, another remarkable result from this paper is that the one-neighbor matching situation has larger increasing rates of errors. This result can be used to help us to understand why we normally select two or more neighbors for the RADAR neighbor matching localization in the practical use (e.g., in [7], the authors choose *K* = 3 to obtain the best accuracy in an indoor straight corridor environment), although the one-neighbor matching situation gives the smallest expected error.

### 4.2. Experimental Results

#### 4.2.1. Environmental Layout

**Figure 9:**
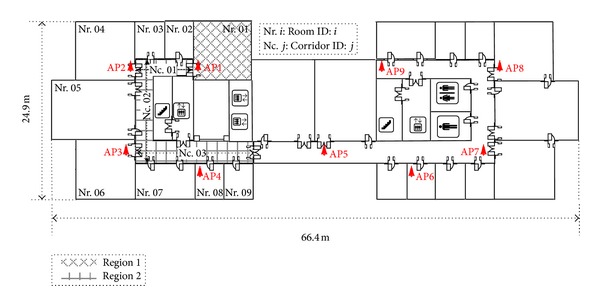
Layout of experimental environment covered by Wi-Fi network.

Figure 9 shows our experimental environment with the dimensions of 66.4 meter × 24.9 meter. In this figure, there are 9 testing Wi-Fi APs (Linksys WAP54G AP) labeled by AP1,…, AP9. All APs are attached to the walls with a height of 2 m from the ground. We choose the nonoverlapped channels (channels 1, 6, and 11) for any three most adjacent APs (i.e., channels for AP1,…, AP9 are the 1, 6, 11, 1, 6, 11, 1, 6, and 11, resp.) to reduce the cochannel interference. The ASUS A8F laptop with the Intel PRO/Wireless 3945ABG network connection wireless card is used as the RSS receiver with the sample rate of 2 Hz. At each RP, we recorded 180 RSS samples to construct the radio map.

**Figure 10:**
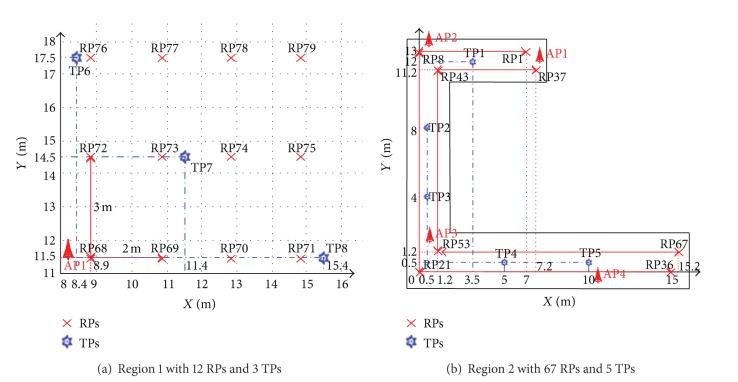
Locations of RPs and TPs in target area.

As can be seen in Figure 9, there are two regions considered for our testing (i.e., region 1 in office Nr. 01 and region 2 in corridors Nc. 01, Nc. 02, and Nc. 03).  Figure 10 shows the locations of RPs and TPs in target area with the origin RP21.

#### 4.2.2. Static Positioning

**Figure 11:**
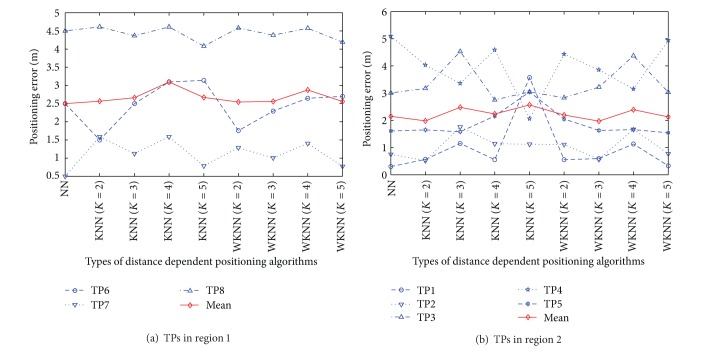
Results of static positioning errors by all the APs.

In the scenario of fixed target positioning, we use the mean value of 60 newly-recorded RSS samples at each TP for the testing. By using all the APs, we compare the static positioning errors between the RADAR neighbor matching localization (i.e., KNN) with *K* = 1 (i.e., NN),…, 5 and the weighted distance dependent positioning (i.e., WKNN) with *K* = 1 (i.e., NN),…, 5 in Figure 11.

**Figure 12:**
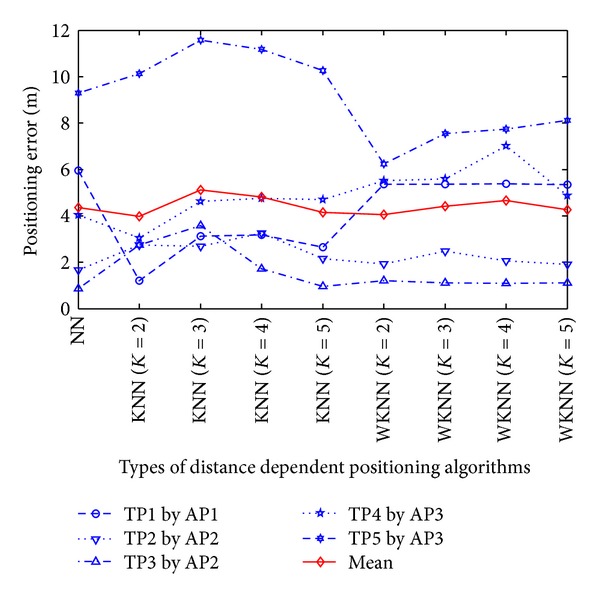
Results of static positioning errors by the strongest AP.

Based on the results in Figure 11, we can obtain the following three observations (i.e., Observations 1, 2, and 3). To illustrate the previous analytical results, in the straight corridors where the RPs are calibrated linearly, we use the AP with the strongest RSS (i.e., the strongest AP) at each TP (i.e., TP1 by AP1, TP2 by AP2, TP3 by AP2, TP4 by AP3, and TP5 by AP3) to calculate the positioning errors, as shown in Figure 12.

**Observation 1**

In the RADAR neighbor matching localization, the NN and KNN with *K* = 2 outperform the KNN with *K* > 2 in terms of the mean of positioning errors. Thus, we can make a conjecture that the increase of the number of neighbors cannot be an effective way to improve the accuracy of RADAR neighbor matching localization.

**Observation 2**

In both the RADAR neighbor matching localization and weighted distance dependent positioning, the positioning accuracy in corridors (i.e., the region 2) is superior to the accuracy in office (i.e., the region 1). For instance, by NN algorithm, the mean of positioning errors is decreased by 12% in corridors compared to the office scenario.

**Observation 3**

For a given number of neighbors, the weighted distance dependent positioning which distributes the different weights to the neighbors performs slightly better than the RADAR neighbor matching localization in terms of accuracy.

As can be seen from Figures 11 and 12, another two observations (i.e., Observations 4 and 5) can be found as follows.

**Observation 4**

In both RADAR neighbor matching localization and weighted distance dependent positioning, the accuracy can be significantly improved with the increase of the number of APs. For instance, NN algorithm reduces the mean of positioning errors in the all-AP scenario by 52% compared to the mean of positioning errors in the strongest-AP scenario.

**Observation 5**

In the strongest-AP scenario, the positioning errors by weighted distance dependent positioning are more stable than the errors by RADAR neighbor matching localization with respect to the variations of the number of neighbors. Therefore, the value of *K* should be carefully studied to explore the highly-accurate RADAR neighbor matching localization system.

#### 4.2.3. Dynamic Positioning

**Figure 13:**
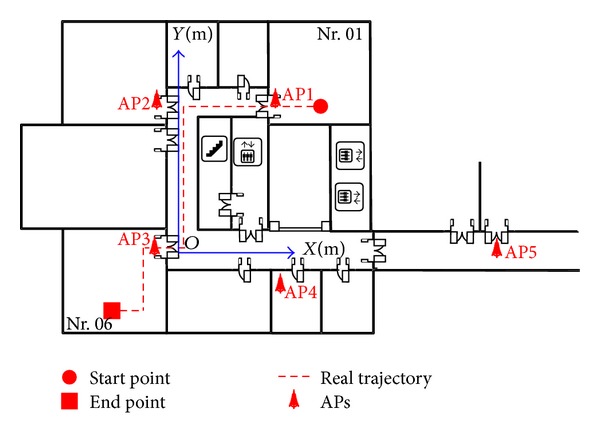
Real trajectory for dynamic positioning in Wi-Fi environment.

Different from the static positioning, both the positioning accuracy and rates should be considered in the dynamic situation. For simplicity, we only focus on the strongest-AP scenario in the following results. The target moves along the real trajectory (see Figure 13) with the speed of 1 meter per second.

**Figure 14:**
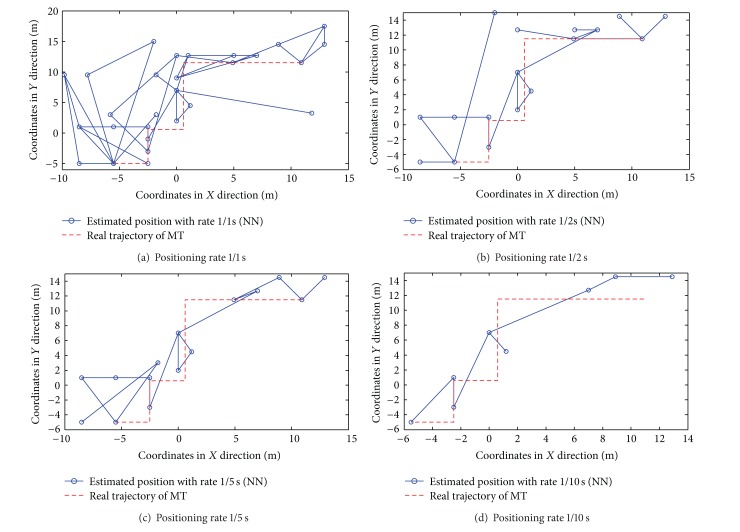
Results of dynamic positioning by NN algorithm.

**Figure 15:**
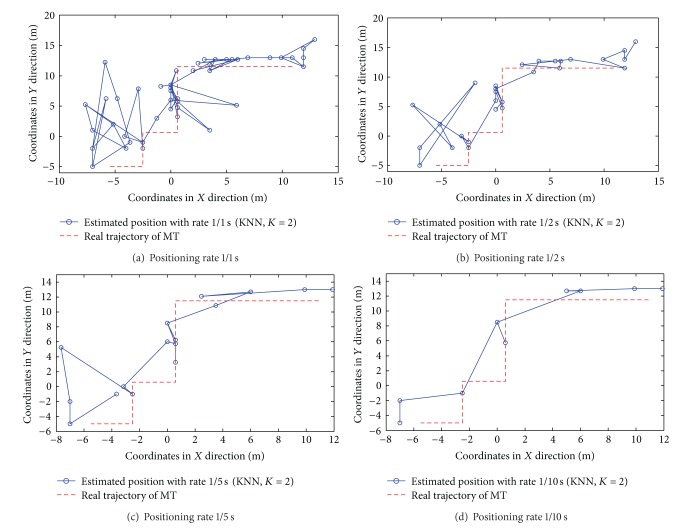
Results of dynamic positioning by KNN with *K* = 2 algorithm.

**Figure 16:**
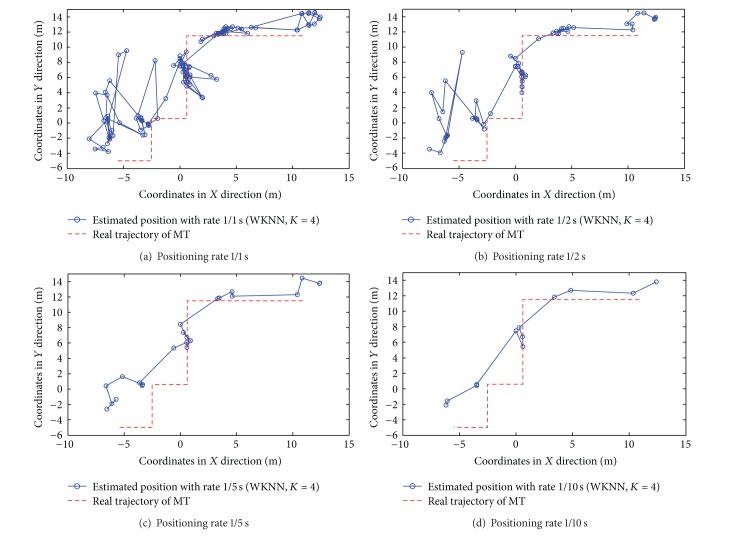
Results of dynamic positioning by WKNN with *K* = 4 algorithm.

The results of dynamic positioning by NN, KNN with *K* = 2, and WKNN with *K* = 4 algorithms are shown in Figures 14, 15, and 16, respectively. The time interval of any two consecutive position calculations is represented by *τ* (*τ* = 1,2, 5, and10) seconds, which means the position estimation is with rate 1/*τ*s.

From the previous figures, the following three observations (i.e., Observations 6, 7, and 8) can be concluded.

**Observation 6**

In both the RADAR neighbor matching localization and weighted distance dependent positioning, the increase of the time interval between any two consecutive position calculations can effectively improve the dynamic positioning accuracy (or the similarity between the connection of estimated positions and the target's real trajectory). In other words, there is a tradeoff between the positioning accuracy and the real-time positioning capability.

**Observation 7**

In both the RADAR neighbor matching localization and weighted distance dependent positioning, the results of dynamic positioning in corridors are more accurate and stable than the positioning results in offices.

**Observation 8**

In the office scenario, the weighted distance dependent positioning slightly outperforms the RADAR neighbor matching localization.

**Figure 17:**
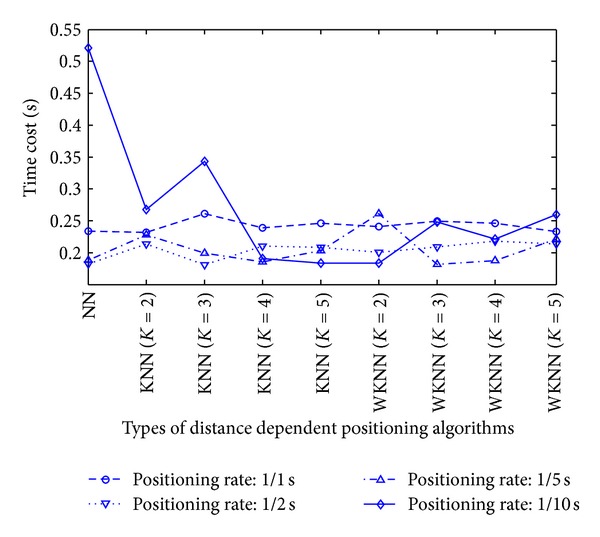
Time cost for each position calculation.

To examine the real-time positioning capability further, we should also consider the time cost involved in each position calculation. In our RADAR neighbor matching localization system, the time cost consists of four main parts as follows: the (i) time cost for the calculation of the average of newly-recorded RSS samples (e.g., the RSS samples in 10 seconds are averaged in the condition of the positioning rate 1/10 s); (ii) time cost for the selection of the parameters corresponding to the smallest statistical error based on the coarse positioning results; (iii) time cost for searching neighbor(s) in radio map; and (iv) time cost for the calculation of the target's estimated position. The time cost for each position calculation by RADAR neighbor matching localization and weighted distance dependent positioning is shown in Figure 17. As seen in Figure 17, the last observation (i.e., Observation 9) is obtained as follows.

**Observation 9**

In the dynamic positioning situation, the NN and KNN with *K* = 2 algorithms with the positioning rate of 1/2 s perform better in the aspects of both the statistical errors and real-time positioning capability.

## 5. Conclusions

This paper has derived out the closed-form solutions to the statistical errors by RADAR neighbor matching localization in a linearly-calibrated RP environment to examine the inherent mathematical relationships between the statistical errors and the number and interval of RPs. The objective of this paper is to explore highly-accurate and cost-efficient neighbor matching localization systems to support ubiquitous LBSs in Wi-Fi environments, through either the judicious deployment of RPs or the parameter optimization in the localization systems. However, due to the existence of RSS interference and distortion in wireless channels, other propagation models (e.g., the logarithmic RSS propagation models containing several break points) should be carefully considered in our ongoing work, and meanwhile the statistical errors with respect to the more generalized models (e.g., the uniformly calibrated RP grids in the 2-dimentional plane area) would also form interesting work in future.

## Figures and Tables

**Table 1:** Statistical errors by RADAR neighbor matching localization.

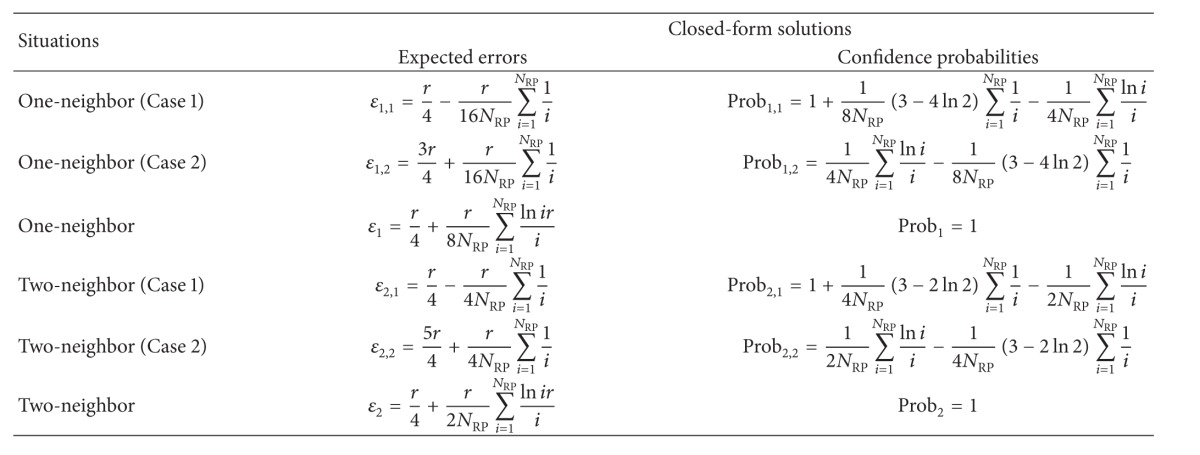

**Table 2:** Variations of the number of RPs and expected errors.

	*r* = 1 m	*r* = 0.8 m	*r* = 0.6 m	Variation rate from *r* = 1 m to 0.8 m	Variation rate from *r* = 1 m to 0.6 m
*N*_RP_	48	60	80	25%	66.7%
*ε*_1_	0.27 m	0.21 m	0.16 m	22.2%	40.7%
*ε*_2_	0.33 m	0.25 m	0.18 m	24.2%	45.5%

## Parameters and Notations

**O**_c_: Origin of coordinates
**A**: AP's position
**T**: TP's real position
*N*_RP_: Number of RPs
*r*: Interval of RPs
*d*_**A**_: Distance between **O**_c_ and **A**
*d*_*i*_ = *ir* (*i* = 1,…, *N*_RP_): Distance between **O**_c_ and **R**_*i*_
*d*_*T*_: Distance between **O**_c_ and **T**
*d*_**T**,1_: Distance between **O**_c_ and **C***by one- neighbor matching
*d*_**T**,2_: Distance between **O**_c_ and **C*** by two-neighbor matching
*d*_**T**,**K**_: Distance between **O**_c_ and **C*** by multineighbor matching
*δ*: Distance between **R**_*i*_ and **T**
*P*_*t*_: AP's transmit power
*P*_*i*_ (*i* = 1, …, *N*_RP_): Mean of received power at **R**_*i*_
*P*_**T**_: Mean of received power at **T**
*E*_*x*_{*f*(*x*)}(*x* = *δ* or *d*_*i*_): Expectation of the *f*(*x*) with respect to the variable *x*
*O*(1/*d*_*i*_)^*κ*^: Higher order term of the (1/*d*_*i*_)^*κ*−1^.
